# A Follow-Up Study of Motor Skill Development and Its Determinants in Preschool Children from Middle-Income Family

**DOI:** 10.1155/2020/6639341

**Published:** 2020-12-15

**Authors:** Huan Wang, Yanjie Chen, Jianing Liu, Huanhuan Sun, Weizhen Gao

**Affiliations:** ^1^China Institute of Sport Science, 100061, China; ^2^Beijing Children's Hospital, Capital Medical University, 100045, China; ^3^Shanghai University of Sport, 200438, China

## Abstract

We tracked the motor skill development of young children aged 3–6 years and investigated the influence of middle-income home environment on the development of motor skill. 268 children were selected from kindergartens in Beijing. The Test of Gross Motor Development (TGMD) tool was used to test the development of locomotor and object-control skills (LS and OS), and a survey of children's behaviour and home environment was conducted. During the follow-up, the LS and OS of children aged 3–6 years continued to grow, with an annual growth rate of 20% and 30%. Five LS indicators and two OS indicators were significantly higher in the 3–4-year group than in the 4–5 and 5–6-year groups (*p* < 0.01). The age-sex trend model showed that girls' locomotor skill developed at a significantly higher rate than that of boys (*β* = 6.3004 and 4.6782, *p* < 0.001). Three-year-old boys performed significantly better than girls on object-control motor skill (*p* < 0.05). Factors affecting the rate of children's motor skill development in middle-income families included the frequency of playing with friends (*β* = 0.133, *p* = 0.032) and the frequency of bicycling, skateboarding, dancing, running, and jumping (*β* = 0.041, *p* = 0.042). Family income, parents' education level, and family activity area did not significantly affect the growth rate of motor skills. For middle-income families, the improvement of material environment at home like more playing spaces and toys did not speed up the motor development, while more opportunities to play with friends and engage in a variety of sports activities could promote children's motor skill development.

## 1. Introduction

Motor skill development plays an important role in children's health and development and is the foundation for performing physical activities [1–3]. The first six years of childhood are devoted to learning and practicing fundamental motor skills in an exploratory and experiential manner, including locomotor skill, object-control skill, and posture-control skill [4]. These motor skills need to be developed at an appropriate level between the ages of three and six, and different types of movements should have their own developmental patterns [5]. Studies on motor development characteristics at the childhood stage have produced diverse results. Previous study found a gradual decrease in the average annual growth value of motor skills with age in a study of 1,614 Belgian children aged 3–6 years [6]. And a study of 1,046 children found that the average annual growth values of locomotor and object-control skills were highest among 4–5 years in children aged 3–6 years [7]. In these studies, the cross-sectional method was used to collect data of different age groups to reflect the changes of the motor development in the process of growth. However, this method has limitation in a certain level. Children in different generations are influenced by different environments; hence, the cross-section method which connects data of different generations is not sufficiently convincing. To gain a deeper understanding of this issue, it is essential to follow up the research data to provide more powerful data support.

In addition to the following physical growth patterns, the development of motor skills is inevitably related to physical activities (PA) [8] and the home environment [9]. Past research reports were inconsistent regarding the relationships between PA and the home environment. Some studies have found no association [10, 11], while others have reported weak but positive association [12, 13]. This could be related to the intensity and type of PA engaged in by the study subjects and with whom they exercise. In addition, young children's activity behaviours depend heavily on the home environment. It was argued that in a family with more abundant sports facilities and a larger space where parents were at a higher educational level, children would be more active and their motor skills would develop faster [14, 15]. However, it has been suggested that the influence of home environment on children's motor skill development is affected by the family's socioeconomic status (SES) and that the key factors affecting children's motor development may differ across families with different SES levels and cannot be generalized [16, 17]. Positive factors that promote motor skill development in low-income families may not necessarily play a role in middle- and high-SES families [18–20]. China is in a period of rapid economic development, and an increasing number of families are moving from low-income to middle-income level. As rapid economic development in China, an increasing number of families have been moved from the low-income group to the middle-income group. It is significant to study the motor development of children in middle-income families and to create a better home sporting environment for their healthy development.

Therefore, this study followed-up the motor skill development of preschool children aged 3–6 years, examined the trajectory of motor and object-control development, and explored the influence of the motor environment on motor development in medium-SES families, aiming at providing a basis for future interventions.

## 2. Methods

### 2.1. Participants

Random cluster sampling was used to select 353 children in 15 classes at four kindergartens in downtown Beijing, of whom 198 were boys and 155 were girls. Ten children with congenital disorders such as motor disorders, intellectual disabilities, Autism Spectrum Disorder, Attention Deficit Hypersensitivity Disorder, and speech disorders were excluded. After consultation with researchers and teachers, parents signed the informed consent form. During the 1-year study period, 75 children were lost from the study because they transferred to other kindergartens or attended preschool. Finally, 268 children, 126 boys (47.0%) and 142 girls (53%), participated in the study: 63 in the 3–4-year group, 127 in the 4–5-year group, and 78 in the 5–6-year group. The characteristic of the participants can be seen in Table 1. The study was approved by the Ethics Committee of China Institute of Sport Science.

### 2.2. Research Procedure

The 268 preschool children were divided into a 3–4-year group, a 4–5-year group, and a 5–6-year group. Two specially trained testers administered the Test of Gross Motor Development-3rd Edition (TGMD-3) test to the subjects from June to July 2017. The entire testing process was videotaped in multiple directions with (model) video camera, and the video was rewatched for any questionable on-site scoring to ensure the consistency of the test results. Moreover, parents were asked to fill out the Environmental Opportunities Questionnaire based on the situation in the past month. From June to July 2018, the same group of testers administered the TGMD-3 test with the same content.

### 2.3. Test Methods

#### 2.3.1. Methods of Motor Development Assessment

Children's motor skill competence was assessed using the TGMD-3. This test was developed by Professor Ulrich in the United States and was used to measure the level of basic motor skill development in children aged 3–10 years, which is considered a valid and reliable instrument [21]. It assesses 13 skills: 6 locomotor (run, gallop, hop, leap, horizontal jump, and slide) and 7 object-control skills (overhand throw, underhand throw, catch, dribble, one-hand strike, two-hand strike, and kick). The total scores for locomotor and object-control skills were 46 and 54, respectively. The testers were college students who received special training before the test. During the test, the testers first demonstrated the correct movements once; following that, the children practiced once and then took two formal tests.

In the quality control of the test, every year, the TGMD test was undertaken by the same college students. Before the test, they were trained with the TGMD-3manual, and they watched videos on the website of the University of Michigan. They tested the children on site and standardized the scores.

The test sequences followed the order from locomotor skill to object control skills and from simple movement to complex movement. However, considering the changes in mood and interest of some children, the test process would be adjusted accordingly, such as starting the test from object control skills.

Before each formal test, 10% of participants were selected to carry out a test-retest reliability experiment. Interrater reliability for locomotor subscale and ball skill subscale was good (ICC: 0.89–0.95). Intrarater reliability across all raters was good (ICC: 0.76–0.96).

#### 2.3.2. Questionnaire

The Environmental Opportunities Questionnaire supporting early motor development in the first year of life is used as a research tool to evaluate the home environment of young children. The scale has good reliability and validity [22], and its Cronbach *α* coefficient used in China is 0.88 [23]. The questionnaire consists of two sections: children's activities and parent questionnaire. It was completed by the guardian after signing an informed consent form. The children's activity section includes the children's health status, siblings' age, leisure sports activities and frequency, existing sports equipment and use, sports activities and frequency after school, and people and partners that children come into contact with in their daily life. The parent questionnaire includes general information about parents (family members, marital status, parents' occupation and educational level, daily caregivers and their educational level, household income per capita, and household living area), parents' participation in sports activities in the last six months, toy supply at home, and parents' attitudes and supportive behaviours towards children's sports (Table 2).

To facilitate the SES assessment of the families of young children, the educational level of the main caregiver, household disposable income per capita, and the household living area were evaluated together. The educational level of the caregiver was scored on a scale of 1–4, in the descending order of graduate, undergraduate, postsecondary and secondary schools, and elementary school. The monthly household income per capita was scored on a scale of 1–4 in a descending order: greater than ¥10,000, ¥5,001–¥100,000, and ¥1,000–5,000 and less than ¥1,000. The household living area per capita was scored on a scale of 1–4 in descending order: greater than 50 m^2^, 31–50 m^2^, and 21–30 m^2^ and less than 20 m^2^. The cumulative score of the three indicators was used as the SES score for young children's families, with a full score of 12; a higher score indicated a better family SES. Based on the results of the 2005 survey conducted by the National Bureau of Statistics of China, the annual income of urban middle-income families in China (calculated based on the average family size of three) was ¥60,000–¥500,000; i.e., the household living area was usually above 80 m^2^. Consequently, SES scores were classified into three grades, with greater than 10 being classified as high-SES families, 6–10 as medium-SES families, and less than 6 as low-SES families.

### 2.4. Statistical Methods

The SPSS18.0 statistical tool was used for statistical analysis of the data. Descriptive statistics were used to describe the TGMD-3 results and questionnaire information for preschool children. The hierarchical linear modelling (HLM) model was used to fit the data from the two longitudinal tests to a developmental curve from age 3–6 years. Age trends and gender differences in the development of locomotor and object-control skills were calculated in turn. *p* < 0.05 was considered statistically significant.

A general linear model (GLM) was used to analyse the effects of age and gender on basic motor skills using a between-subjects effect test and parameter estimation with gender and age as fixed factors, with the average annual growth value of each item as the dependent variable. Finally, a multifactor generalized linear regression analysis was conducted with the annual growth rate (%) of the TGMD-3 score as the dependent variable and age, individual activity behaviour, and family support environment as the independent variables, to explore the factors influencing the rate of motor skill development. The independent variables with the highest *p* values were eliminated one by one using the regression method, and the independent variables were retained according to *α* ≤ 0.05, thus building the final model. Statistical significance was set at *p* < 0.05.

## 3. Results

### 3.1. Annual Growth Rate of Motor Skills

Table 3 shows that boys and girls in the three age groups improved their locomotor and object-control skills after one year of growth. The average annual growth in locomotor skills was 3.9 points for boys and 6.0 points for girls, while the average annual growth in object-control skills was 5.8 points for boys and 5.6 points for girls. Dividing the TGMD average annual growth value by the baseline value to the average annual growth rate resulted in an annual growth rate of 14.7–22.2% for locomotor skills and 30.6–31.7% for object-control skills.

**Figure 1:**
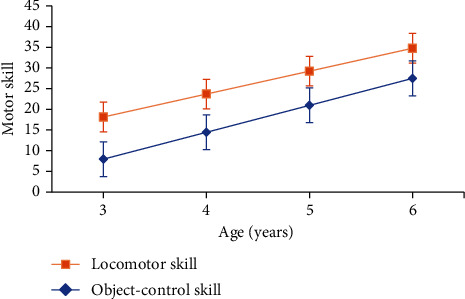
Age characteristics of locomotor and object-control skills.

The one-year motor skill growth of the three age groups was used to fit the motor skill change curves from three to six years old. Taking children's locomotor skills and object-control skills as the dependent variables and age as the independent variable, a trend model of motor skill level and age (HLM model) was constructed to describe the trends in children's locomotor skills and object-control skills with age. The convergence test showed that the models all conformed to the principle of convergence; therefore, the development trend of children in each age group was basically consistent and could be described by a single curve. The slopes of the fixed parts of the locomotor and object-control scores were statistically significant (*p* < 0.001), indicating that both scores changed linearly with age and that the slope of object-control skills was larger than that of locomotor skills.  Figure 1 shows the resulting age-specific change curves.

**Figure 2:**
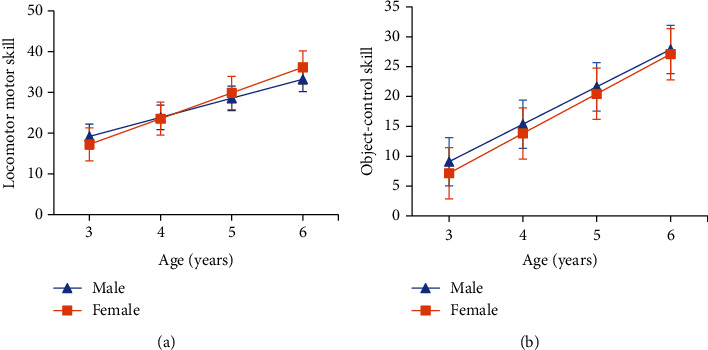
Gender characteristics of locomotor and object-control skills: (a) locomotor skill; (b) objective-control skill.

Gender was included in the model to further investigate whether there were gender differences in the age-dependent characteristics of children's locomotor and object-control scores, and the results are shown in Figure 2.  Figure 2(a) shows that at the age of 3 years, boys' locomotor skills was slightly higher than that of girls, but there was no statistical difference (*p* > 0.05). However, girls' locomotor skills developed at a significantly higher rate with age than that of boys (*p* < 0.001), with slopes of 6.3004 and 4.6782, respectively.  Figure 2(b) shows that at the age of three years, boys' object-control skills was significantly higher than that of girls (*p* < 0.05), but girls' locomotor skills developed at a significantly higher rate with age than that of boys (*p* < 0.001), with slopes of 6.6485 and 6.2663, respectively (*p* < 0.001).

### 3.2. Influence of Age and Gender on the Growth Rate of Motor Skills

The GLM was used to further examine the effects of age and gender on the results of the 13-item TGMD motor indicators (Table 4). A between-subjects effect test and parameter estimation were conducted using the average annual growth value of each item as the dependent variable, with gender and age group as fixed factors.

Of TGMD's 13 locomotor and object control skills, gender had a significant effect on the growth rate of gallop performance, with girls having significantly higher average annual growth values than boys (*p* = 0.01, *β* = −1.07). In the six locomotor skill indicators, age had a significant effect on running, gallop, slide, horizontal jump, and hop. The average annual growth value was significantly higher in the 3–4-year group than in the 4–5-year group and 5–6-year group (*p* < 0.01), with no significant difference between the two groups. The effect of age on the average annual growth value of object-control skills was related to the indicator. The table shows that the skill growth of overhand throw (*β* = 1.25, *p* = 0.01) and kick (*β* = 0.10, *p* = 0.02) was age related, with the fastest average annual growth in the 3–4-year group. Growth in other ball skills did not differ significantly between age 3, 4, and 5 years (*p* > 0.05) (Table 4).

### 3.3. Individual Behaviour and Home Environment of the Children

In the first year of the baseline test, the children's individual behaviour and home environment were investigated. The survey showed that there were only significant differences between diverse age groups in the frequency of using sports equipment and that there were no age differences in other behavioural indicators (*p* < 0.05). The SES level of children's families was relatively concentrated in the range of 8.7–8.9 points, which belonged to medium-SES families. In the parents' support for children's physical activities, the highest degree of “entirely supportive” was for “chaperoning and transportation,” reaching over 60%. The proportion of parents participating in physical activities more than 3 times a week was about 30% (Table 5).

### 3.4. Influence of Young Children's Behaviour and Home Environment on the Growth Rate of Motor Skills

In this section, a multifactorial GLM regression analysis was conducted using the annual growth rate (%) of TGMD scores as the dependent variable and children's age, individual activity behaviour, and family support environment as the independent variables, to explore the factors influencing the rate of motor skill development.

Using the regression method, the independent variables with the highest *p* values were eliminated one by one in sequence, and the independent variables were retained according to the principle of *α* ≤ 0.05, thereby building the final model (Table 6). The model suggested that children's age (*β* = −0.393, 95% CI: 0.552~-0.234), the weekly frequency of sports activities (*β* = 0.133, 95% CI: 0.021~-0.245), and the number times of playing with friends (*β* = 0.041, 95% CI: 0.001~-0.081) were associated with the speed of motor skill development. According to the regression coefficients and their significance tests, the annual growth of TGMD score was faster in children at a younger age, with a higher frequency of sports activities, and in a greater number times of playing with friends. Conversely, other behavioural factors such as the frequency of media activities and the frequency of use of sports equipment were not associated with the rate of motor skill development. Three family factors (family SES, parental support for sports activities, and parental physical activities level) did not significantly affect the growth rate of motor skills.

Although the model constructed in the last step (*F* = 10.505), was significant, the coefficient of determination and adjusted coefficient of determination were low (*R*^2^ = 0.175 and 0.158, respectively). This suggested that there might have been other crucial influencing factors that have not been included in the equation and await exploration in future studies.

Based on the speed of motor skill development, children with annual TGMD change rate ≥ 50% and ≤5% were selected to compare daily activity behaviour and home support environment, and the results are shown in Table 7. The two groups significantly differed in the weekly frequency of sports activities and the number of times of playing with friends (*p* < 0.05). There were differences in home environment scores, with children with fast motor skill development having lower family SES scores than those with slow motor skill development, but they were not statistically significant. The proportion of parents who were “entirely supportive” of children's sports activities was slightly higher in children with fast motor skill development, as well as the proportion of parents exercising more than 3 times a week, but the overall difference was not statistically significant.

## 4. Discussion

The golden age for motor development is between the ages of three and six years. Understanding the trends and characteristics of motor development is a prerequisite for formulating targeted development strategies. We tracked and analysed the trajectory of the gross motor development of children from urban middle-income families for one year. We identified the characteristics of children's motor development in different motor categories, ages, and genders and compared the effects of different activities and family support environments on children's motor skill development. The results will provide empirical evidence for preschool education workers and parents to guide children to develop motor skills reasonably.

### 4.1. Development Characteristics of Motor Skills by Age

Our results showed that after one year of natural growth, children aged three to six years developed both locomotor and object-control skills each year. The annual growth score of locomotor skills was higher than that of object-control skills, whereas the annual growth rate of locomotor skills was greater. This was consistent with a previous study which is conducted in Xi'an, China [24]. Children's basic motor development follows the principle of improving from simple movement to complex movement and from low-level skill to high-level skill [25]. From 0 to 3 years old, children's locomotor skills are mainly exercised. Their muscles, bones, and cardiopulmonary function are not fully developed, and the main exercise is crawling, walking, and running. After 3 years old, children's gross motor development has already had a certain foundation. They are no longer satisfied with simple walking, running, and jumping and began to develop coordination and object-control skills while consolidating locomotor skills. By fitting the change curves of locomotor and object-control skills, we found that the growth rate of 3 to 6-year-old children's object-control skills was faster than that of locomotor skills. It indicated that as more complicated skills, the object-control skills might develop slower than the locomotor skills in the initial stage [26], but as the nervous system matures, the development of object-control skills would accelerate and may even exceed that of the locomotor skills. Therefore, after the age of 3, appropriate ball games should be provided to the children for promoting their object-control skills.

### 4.2. Influence of Gender on Motor Skill Development

This study indicated that there were gender differences in motor skill development in early childhood. Particularly, girls outperformed boys in locomotor skill development in early childhood, which was similar to the results of Legear et al. and Kit et al. [27, 28]. Nevertheless, some studies have not shown gender differences in the acquisition of some locomotor skills [6, 29]. The research showed that there was no significant difference in biological maturity between boys and girls in early childhood, so their motor skill developments were similar [27]. However, in addition to biological maturity, the motor development of boys and girls is also affected by the social and family environment [30, 31]. It has been demonstrated that girls are more cooperative and sharing in instructional interactions and games, watching action performance, and cheering each other on, whereas boys are more self-expressive and competitive [32]. Thus, for locomotor skills that are relatively competitive and less difficult to perform, girls perform better than boys on some movements. In our test of six locomotor skills, girls performed significantly better than boys on gallop. The reason might be that girls would have more opportunities of dancing, so they have better sense of rhythm [6, 7, 33].

Conversely, the development of object-control skills in early childhood was superior for boys over girls, which was similar to most current research results [6, 27, 28]. The reasons are threefold: first, there are gender differences in the strength of boys and girls, and pitching among object-control movements is particularly pronounced as the movement most related to strength [30]. Second, boys master object-control skills faster than girls and can approach the mature movement stage earlier, a phenomenon that is particularly evident in technically difficult movements such as pitching [34, 35] and kick [36, 37]. The third reason is related to sociology. As noted earlier, boys place a greater value on individual performance and competition among peers and are more willing to improve their technical skills, leading to more practice in this area than girls and more effective improvement of their object-control skills [30].

### 4.3. Influence of Age on Motor Skill Development

Our results showed that age had a significant effect on the average annual growth value of locomotor skills for children aged 3–6 years, showing a gradual decrease in the average annual growth value with age. The average annual growth rate of object-control skills was the same. However, basic object-control skills, kick and overhand throw, grew faster at the age of 3–4 than at the age of 4–5 and 5–6. Kick and pitching are common movements used by children in everyday ball play and require some muscle strength and coordination of the limbs. These two ball skills are easier to master than other object-control skills [38]. According to the principle of simple to complex development of basic movements [25], these two motor skills will develop earlier. Therefore, at the age of 3–4, in addition to focusing on locomotor skills, the learning of ball skills can start with kick and overhand throw.

A study found a significant effect of age on both locomotor and object-control skills in a cross-sectional study of 1,614 Belgian 3- to 6-year-old children, and the average annual growth value of locomotor skills decreased with age [6]. In a cross-sectional study of basic motor skill development in 1,046 children aged 3–10 years in Shandong, China, it was suggested that the average annual growth values of both locomotor and object-control skills were highest in children aged 4–5 years, followed by those aged 3–4 years and 5–6 years [7]. In a cross-sectional study of basic motor skill development in 1,200 preschool children aged 3–7 years in Taiwan, it was revealed that age had a significant effect on both locomotor and object-control skills, and the average annual growth values of both locomotor and object-control skills were highest in children aged 4–5 years, followed by those aged 3–4 years and 5–6 years [27]. Their findings were not exactly the same as ours, which may be due to the following:

1. Varying research design: other studies tended to adopt a cross-sectional design and used different children for the analysis of annual rates of change. In contrast, our study was a longitudinal follow-up study, in which the same subjects were used to calculate the annual growth value and rate. Thus, the data from the follow-up study may be more convincing than those from the cross-sectional study.
2. Varying ethnicity and geographic location of the study subjects: our subjects were preschoolers in Beijing, China, while other studies have reported on mostly Western children [6, 7, 33, 39]. Whether the developmental characteristics of motor skills in young children are related to ethnicity and geography has not yet been reliably evidenced, and further research is needed.

### 4.4. Influence of Young Children's Activity Behaviour and Home Environment on Motor Skill Development

After one year of follow-up, our findings revealed that the acquired factors affecting motor skill development were mainly the amount of physical activities and the frequency of playing with friends, while family SES, parental support, and parents' own exercise level did not have a significant effect.

The survey data exhibited that the physical activities that young children frequently participated in were bicycling, skateboarding, roller skating, dabbling, swimming, dancing, patting, kick, running, and jumping. A higher frequency of daily physical activities leads to faster motor skill development. Especially at the age of 3–4 years when motor skills are developing rapidly, it is necessary to participate more frequently in these sports. The literature is inconsistent regarding the relationship between motor skill levels and physical activities: some authors suggest a strong correlation with moderate-intensity activities, while E Kipling Webster argues that TGMD scores in 3–5 years are only related to high-intensity physical activities [40] and not to moderate-intensity physical activities. More high-intensity activities result in higher TGMD scores [41]. Nilsen AKO's findings support E Kipling Webster that only high-intensity physical activities (accelerometer counts of 5,000–8,000 cpm) are associated with TGMD [42]. In addition to measuring the amount and intensity of physical activities, some scholars have also studied the relationship between the type of physical activities and motor skills of young children. It is believed that preschool children's participation in targeted sports, such as running, bicycling, skateboarding, and trampolining, is associated with higher TGMD scores [43]. Similar to the results of the present study, when participating in these sports, children's physical activity level is high, and they also develop locomotor-type motor skills and ball control skills in running, jumping, and playing with a ball, laying the foundation for future participation in more complex sports.

This study also found that children growing up to play more and exercise more with their peers had an accelerated rate of development of motor skills. Scholar Deanne Fay compared the effects of individual play environments and multiperson group play environments on children's motor skill development. Repeated measures of within-group statistics showed that children performed better on motor skill assessment in group settings, which was associated with being influenced by competition, motivation, and role modelling when playing with multiple people [44]. Hung and Pang carried out a comparison of the effects of group and individual interventions for young children with motor developmental delays. The results showed that although both approaches improved motor skills, activities with 4–5 children were more popular, compliance was better, and the program was more cost-effective overall [45]. In China, one-child families are the majority; thus, children do not have siblings as companions at home and spend most of their time playing with their parents and grandparents, which lacks the supportive peer effect. Therefore, parents should take their children out of the home more often to play with their neighbours and friends in the playground, to learn motor skills, enrich motor experience, cultivate motor interest, and develop motor skills through an interactive play.

According to Barnett et al.'s study, in addition to the children's own activities, home environmental factors are also associated with motor skill development, and the activity behaviour of 3–6 years depends largely on the home environment and parenting style [46]. However, in the present study, an analysis through the GLM model showed that family support for children's exercise and parents' own exercise level did not affect the speed of motor skill development. The reasons for this were two-fold: first, the influence of family on children's motor skill development in this study was reflected through parental self-evaluation, and the subjectivity of responses could affect the accuracy of the analysis of the influence relationship. In addition, the family SES of the young children in this study was medium; i.e., the household income per capita was between ¥5,000 and ¥10,000, the parents' education level was college or higher, and the material conditions of the families were similar. A lack of comparison with low-income families made it difficult to demonstrate the influence of different household living areas as well as sports equipment supply and use on the motor skill development of young children. Consequently, this study did not produce the same results of other studies on the influence of family SES on the development of motor skills in young children [47]. However, by comparing the behavioural characteristics and home environment of children with fast and slow motor development, this study found clues about the relationship between different middle-income families and children's motor development, although it did not show statistical significance. In middle-income families, the children's motor development was slower under better family conditions (SES score: 8.9). The proportion of parents with a graduate degree in these families reached 23%, their support for children's sports was lower than that in families with faster motor development (SES score 8.2), and the children exercised and played less frequently with their friends. It has been indicated that highly educated Chinese parents attach great importance to their children's early education, especially in reading, mathematics, and art and do not want to occupy a lot of time for their children to play freely with friends [48, 49], which may affect their children's early motor skill development to some extent. Whether these subtle differences between middle-income families are truly environmental factors that influence the rate of motor skill development in young children, however, needs to be further confirmed by more convincing research data.

The following are the strengths and limitations: the first strength of this study lies in its use of a tracking approach to study the developmental characteristics of motor skills in the early years of children aged 3–6 years, and the persuasive power of the data is better than the evidence provided by previous cross-sectional studies. The second strength is that it provides an in-depth analysis of the individual behaviours and home environmental factors that influence children's motor development in China's emerging middle-class families, which provides an empirical basis for preschool education workers and parents to guide children's motor skill development. The first limitation of this study is that the follow-up period is only one year; a longer follow-up period will provide a better understanding of the pattern of motor development in Chinese children. The second limitation is the use of an internationally used questionnaire for the survey of family support environment. Since Chinese family education has its own culture and national conditions, it is necessary to develop a Chinese family survey that includes both objective and subjective environmental factors, thereby fully exploring the appropriate ecological patterns that influence children's motor development.

## 5. Conclusions

Locomotor and object-control skills continue to grow between the ages of 3 and 6 years, with object-control skills growing faster than locomotor skills. With the age group of 3–4 years having the fastest growth rate, it is recommended to practice more basic movements such as running, jumping, overhand throw, and kick [50]. Boys and girls between the ages of 3 and 6 also have their own gender advantages in motor skill development, and therefore, priorities can be considered accordingly in their practice activities.

Factors influencing children's motor skill development in Chinese middle-income families include doing sports with friends, bicycling, skateboarding, dancing, bouncing ball, kicking ball, running, jumping, etc.; however, the material sporting conditions offered by families have no influence on children's motor skill development. How families provide effective support for children's motor skill development needs further research and exploration.

## Figures and Tables

**Table 1:** Height, weight, and BMI of the children at the beginning of the study.

Indicator	Age					
	3-4 yr. (*n* = 63)		4-5 yr. (*n* = 127)		5-6 yr. (*n* = 78)	
	Mean	SD	Mean	SD	Mean	SD
Height (cm)	103.6	5.1	107.4	5.3	113.1	5.1
Weight (kg)	16.9	2.9	18.0	2.5	20.1	3.2
BMI (kg/m^2^)	15.7	1.6	15.6	1.3	15.6	1.5

**Table 2:** Key indicators of the Environmental Questionnaire.

Variable category	Indicator	Assignment description
Children behaviour	Weekly frequency of sports activities	Continuous variable, cumulative frequency of multiple physical activities, including bicycling, skateboarding, roller skating, swimming, and playing ball
	Weekly frequency of media activities	Continuous variable, cumulative frequency of multiple media activities, including watching TV, videos, DVDs, and playing video games
	Frequency of use of sports equipment	Continuous variable, cumulative frequency of use of multiple sports equipment, including balls, skates, bicycles, swings, slides, and skipping ropes
	Number of times of playing with friends	More than 5 times per week 3–5 times per week 1–3 times per week Occasionally Never

Home environment	SES	Continuous variable, a higher score indicates a better family SES.
	Parental support for children's sports activities (i) We do a lot of physical exercises with our children (ii) Our family values sports (iii) As parents, we are very interested in our children's sports activities (iv) Our children's sports activities are often a topic of conversation in our family (v) As parents, we support our children's sports activities (e.g., chaperoning and transportation).	Entirely supportive Basically supportive Uncertain Basically unsupportive Entirely unsupportive
	Frequency of parents' physical exercise	More than 5 times per week 3–5 times per week 1–2 times per week No participation

**Table 3:** TGMD test scores of motor skills.

Test item	Baseline test		Testing after one year		Annual growth value						Annual growth rate (%)	
	Male	Female	Male	Female	Male			Female			Male	Female
					Average annual growth value	95% CI		Average annual growth value	95% CI			
	Mean ± SD	Mean ± SD	Mean ± SD	Mean ± SD		Lower bound	Upper bound		Lower bound	Upper bound		
Run	6.33 ± 1.93	6.19 ± 2.14	7.00 ± 1.56	7.01 ± 1.26	0.67	0.26	1.09	0.82	0.41	1.22	10.70	13.20
Gallop	4.71 ± 2.61	4.26 ± 2.72	4.94 ± 2.74	5.55 ± 2.21	0.22	-0.46	1.09	1.29	0.76	1.82	4.70	30.20
Side	6.04 ± 2.36	5.98 ± 2.57	6.33 ± 2.02	6.86 ± 1.66	0.29	-0.20	0.78	0.88	0.41	1.35	4.90	14.70
Skip	2.51 ± 2.20	3.01 ± 2.21	3.45 ± 2.16	4.07 ± 2.13	0.94	0.44	1.45	1.06	0.63	1.49	37.70	35.40
Horizontal jump	4.29 ± 1.73	4.27 ± 1.93	4.98 ± 1.89	5.30 ± 1.80	0.69	0.23	1.15	1.04	0.63	1.44	16.10	24.30
Hop	3.04 ± 2.19	3.45 ± 2.00	4.17 ± 2.25	4.39 ± 2.20	1.13	0.63	1.64	0.94	0.51	1.38	37.30	27.30
Overhand throw	3.12 ± 2.05	2.89 ± 2.00	4.16 ± 1.86	3.83 ± 1.78	1.04	0.55	1.53	0.94	0.55	1.33	33.30	32.70
Underhand throw	2.40 ± 1.88	2.13 ± 1.55	3.14 ± 1.77	2.85 ± 1.83	0.74	0.34	1.14	0.71	0.38	1.04	30.70	33.30
Catch	2.93 ± 1.57	2.89 ± 1.68	3.40 ± 1.68	3.50 ± 1.86	0.47	0.09	0.85	0.61	0.26	0.95	16.00	20.90
Dribble	1.99 ± 2.13	2.16 ± 2.27	3.46 ± 2.31	3.40 ± 2.21	1.47	0.98	1.96	1.24	0.82	1.66	73.70	57.30
One-hand forehand strike	0.95 ± 1.29	0.83 ± 1.17	1.95 ± 1.86	1.45 ± 1.76	1.00	0.61	1.39	0.62	0.27	0.97	105.00	74.60
Two-hand strike	4.10 ± 2.39	3.51 ± 2.49	3.94 ± 2.62	3.83 ± 2.85	-0.17	-0.72	0.38	0.32	-0.21	0.84	-4.10	9.00
Kick	3.56 ± 1.53	3.50 ± 1.47	4.84 ± 1.91	4.77 ± 1.83	1.29	0.87	1.70	1.27	0.90	1.65	36.20	36.40
Locomotor skills	26.91 ± 9.17	27.15 ± 9.80	30.87 ± 7.78	33.18 ± 6.64	3.96	2.05	5.87	6.03	4.49	7.56	14.70	22.20
Object-control skills	19.06 ± 8.69	17.94 ± 8.56	24.89 ± 9.10	23.63 ± 9.14	5.83	4.01	7.66	5.69	4.31	7.07	30.60	31.70
Overall	45.97 ± 16.34	45.10 ± 16.92	55.76 ± 7.62	56.82 ± 6.89	9.79	6.53	13.06	11.72	9.34	14.09	21.30	26.00

SD, Standard deviation; CI, Confidence interval.

**Table 4:** The effects of age and gender on the results of TGMD 13 indicators based on the GLM model.

Test item	Groups	Mean square	*F*	Sig.	*β*	95% confidence interval		Sig.
						Lower bound	Upper bound	
Run	Gender = male	1.03	0.19	0.67	-0.12	-0.69	0.45	0.67
	Gender = female				0^a^			
	Age group = 3‐4	28.83	5.18	**0.00** ^∗∗^	1.21	0.43	2.00	**0.00** ^∗∗^
	Age group = 4‐5				0.23	-0.44	0.90	0.50
	Age group = 5‐6				0^a^			

Gallop	Gender = male	76.37	6.18	**0.01** ^∗^	-1.07	-1.92	-0.22	**0.01** ^∗^
	Gender = female				0^a^			
	Age group = 3‐4	25.90	2.10	0.13	1.20	0.03	2.38	**0.04** ^∗^
	Age group = 4‐5				0.68	-0.32	1.68	0.18
	Age group = 5‐6				0^a^			

Slide	Gender = male	19.17	2.63	0.11	-0.54	-1.19	0.12	0.11
	Gender = female				0^a^			
	Age group = 3‐4	77.71	10.67	**0.00** ^∗∗^	1.66	0.76	2.56	**0.00** ^∗∗^
	Age group = 4‐5				-0.20	-0.97	0.57	0.61
	Age group = 5‐6				0^a^			

Skip	Gender = male	0.81	0.11	0.74	-0.11	-0.77	0.55	0.74
	Gender = female				0^a^			
	Age group = 3‐4	1.15	0.15	0.86	0.09	-0.82	1.00	0.84
	Age group = 4‐5				-0.13	-0.91	0.64	0.74
	Age group = 5‐6				0^a^			

Horizontal jump	Gender = male	6.82	1.11	0.29	-0.32	-0.92	0.28	0.29
	Gender = female				0^a^			
	Age group = 3‐4	21.51	3.50	**0.03** ^∗^	0.91	0.08	1.74	**0.03** ^∗^
	Age group = 4‐5				-0.06	-0.76	0.65	0.88
	Age group = 5‐6				0^a^			

Hop	Gender = male	2.71	0.37	0.55	0.20	-0.46	0.86	0.55
	Gender = female				0^a^			
	Age group = 3‐4	21.97	2.97	0.05	1.10	0.19	2.01	0.01^∗^
	Age group = 4‐5				0.33	-0.44	1.10	0.40
	Age group = 5‐6				0^a^			

Overhand throw	Gender = male	0.57	0.09	0.76	0.09	-0.52	0.70	0.76
	Gender = female				0^a^			
	Age group = 3‐4	27.62	4.35	**0.01** ^∗^	1.25	0.41	2.09	**0.00** ^∗∗^
	Age group = 4‐5				0.68	-0.04	1.39	0.06
	Age group = 5‐6				0^a^			

Underhand throw	Gender = male	0.09	0.02	0.89	0.04	-0.48	0.55	0.89
	Gender = female				0^a^			
	Age group = 3‐4	3.53	0.78	0.46	0.38	-0.34	1.09	0.30
	Age group = 4‐5				-0.01	-0.62	0.59	0.97
	Age group = 5‐6				0^a^			

Catch	Gender = male	1.40	0.31	0.58	-0.15	-0.66	0.37	0.58
	Gender = female				0^a^			
	Age group = 3‐4	3.55	0.79	0.45	-0.41	-1.12	0.29	0.25
	Age group = 4‐5				-0.05	-0.65	0.55	0.86
	Age group = 5‐6				0^a^			

Dribble	Gender = male	4.02	0.57	0.45	0.25	-0.40	0.89	0.45
	Gender = female				0^a^			
	Age group = 3‐4	11.66	1.65	0.20	0.70	-0.19	1.58	0.13
	Age group = 4‐5				0.00	-0.76	0.75	1.00
	Age group = 5‐6				0^a^			

One-hand forehand strike	Gender = male	9.68	2.04	0.16	0.38	-0.15	0.91	0.16
	Gender = female				0^a^			
	Age group = 3 ~ 4	3.67	0.77	0.46	-0.46	-1.18	0.27	0.22
	Age group = 4 ~ 5				-0.24	-0.86	0.37	0.44
	Age group = 5 ~ 6				0^a^			

Two-hand strike	Gender = male	14.76	1.48	0.23	-0.47	-1.23	0.29	0.23
	Gender = female				0^a^			
	Age group = 3‐4	2.83	0.28	0.75	0.20	-0.85	1.26	0.70
	Age group = 4‐5				-0.16	-1.06	0.73	0.72
	Age group = 5‐6				0^a^			

Kick	Gender = male	0.16	0.03	0.86	0.05	-0.50	0.60	0.86
	Gender = female				0^a^			
	Age group = 3‐4	20.12	3.87	**0.02** ^∗^	0.10	-0.66	0.86	0.80
	Age group = 4‐5				-0.73	-1.38	-0.09	0.03
	Age group = 5‐6				0^a^			

Overall locomotor	Gender = male	255.82	2.69	0.10	-1.96	-4.32	0.40	0.10
	Gender = female				0^a^			
	Age group = 3‐4	786.35	8.26	**0.00** ^∗∗^	6.18	2.92	9.43	**0.00** ^∗∗^
	Age group = 4‐5				0.85	-1.92	3.62	0.55
	Age group = 5‐6				0^a^			

Overall object-control	Gender = male	3.01	0.04	0.85	0.21	-2.03	2.46	0.85
	Gender = female				0^a^			
	Age group = 3‐4	112.19	1.29	0.28	1.75	-1.36	4.85	0.27
	Age group = 4‐5				-0.55	-3.19	2.09	0.68
	Age group = 5‐6				0^a^			

Overall performance	Gender = male	203.31	0.78	0.38	-1.75	-5.64	2.15	0.38
	Gender = female				0^a^			
	Age group = 3‐4	1444.07	5.54	**0.00** ^∗∗^	7.92	2.54	13.31	**0.00** ^∗∗^
	Age group = 4‐5				0.30	-4.28	4.88	0.90
	Age group = 5‐6				0^a^			

^a^Reference. The bold font is used to highlight significance level at *p* < 0.05. ^∗^*p* < 0.05; ^∗∗^*p* < 0.01.

**Table 5:** The behaviour and home environment of the participants.

Category	Indicator (mean ± SD or %)	3 years	4 years	5 years
Children behaviour	Weekly frequency of sports activities	11.0 ± 2.7	11.0 ± 2.6	12.0 ± 2.7
	Weekly frequency of media activities	14.0 ± 2.8	13.0 ± 3.1	15.0 ± 3.0
	Frequency of use of sports equipment	14.0 ± 2.4	14.0 ± 2.0	18.0 ± 2.5
	Number of playdates with friends	10.0 ± 1.9	10.0 ± 2.3	9.0 ± 2.4

Family SES	Parental educational level Household disposable income per capita household living area	8.7 ± 1.5	8.8 ± 1.9	8.8 ± 1.9

Parental support for children's sports activities (full support ratio %)	As parents, we accompany and transport children to participate in sports.	63.4%	75.5%	66.7%
	We do a lot of physical exercises with our children.	22.5%	17.1%	16.0%
	Our family values sports.	30.1%	36.1%	38.0%
	As parents, we are very interested in our children's sports activities.	37.5%	47.2%	48.1%
	Our children's sports activities are often a topic of conversation in our family.	28.9%	36.8%	25.2%

Proportion of parents' physical exercise level	More than 3 times a week	30.8%	34.6%	28.3%

SD, Standard deviation.

**Table 6:** Final GLM model and parameter estimation.

Indicator	Mean square	*F*	Sig.	*β*	95% confidence interval	
					Lower bound	Upper bound
Constant	11.292	28.040	0.000	2.419	1.516	3.322
Age	9.577	23.781	0.000	-0.393	-0.552	-0.234
Weekly frequency of sports activities	2.095	6.023	0.032	0.133	0.021	0.245
Number times of playing with friends	1.687	4.189	0.042	0.041	0.001	0.081

*R* ^2^ = 0.175 (adjusted *R*^2^ = 0.158).

**Table 7:** Comparison of activity behaviour and home environment of children with the different speeds of motor skill development.

	Fast motor skill development (annual growth value of TGMD ≥50%)	Slow motor skill development (annual growth value of TGMD ≤5%)	P
Sample size	66	70	
Weekly frequency of sports activities	12.100 ± 2.800	10.600 ± 2.100	0.028
Number times of playing with friends	3.300 ± 1.000	2.100 ± 1.200	0.045
Family SES score	8.290 ± 1.750	8.940 ± 1.790	0.152
Parental support			0.163
Entirely supportive	13.6%	6.7%	
Basically supportive	59.3%	70.7%	
Uncertain	25.4%	20.0%	
Basically unsupportive	1.7%	2.7%	
Average parental exercise level			0.231
More than 3 times a week	20.3%	15.4%	
1-2 times a week	44.9%	49.2%	
Less than once a week	39.7%	28.8%	

## Data Availability

The data used to support the findings of this study are available from the corresponding author.
